# A Bad Case

**Published:** 1874-04

**Authors:** 


### A BAD CASE.

The following lucid statement of his case was lately forwarded by a patient to his medical attendant.:—

"I have a very bad stomach and sickness about my hart and great heat rising up true me and sweeting in my face and at the but of the troth (throat) alys stifliing me and all the trouble of the wourrld in it and very bound in the bouls and a pain in my head, and i douse allways be incline to discarge my stomach, and i never can, and i have often a great griping and a great bast (?) in my lung, and i dose bi belshing up every minut."

